# Howard’s Method of Artificial Respiration

**Published:** 1881-09

**Authors:** 


### HOWARD'S METHOD OF ARTIFICIAL RESPIRATION.

*Quickly* turn the patient's face upward, with a roll of clothing under the back, just below the shoulder blades, and make the head hang back as low as possible. Place the patient's hands above his head. Kneel with the patient's hips between your knees, and fix your elbows firmly against your hips. Now, grasping lower part of the patient's naked chest, squeeze his two sides together, pressing *gradually* forward with all your weight, for about three seconds, until your mouth is nearly over the mouth of patient; then, with a push, *suddenly* jerk yourself back. Rest about three seconds; then begin again, re-peating these bellows-blowing movements with perfect regularity, so that foul air may be pressed out and pure air be drawn into the lungs, about eight or ten times a minute, for at least an hour, or until the patient breathes naturally.

